# Use and Effectiveness of Antimicrobial Intravesical Treatment for Prophylaxis and Treatment of Recurrent Urinary Tract Infections (UTIs): a Systematic Review

**DOI:** 10.1007/s11934-018-0834-8

**Published:** 2018-08-09

**Authors:** Amelia Pietropaolo, Patrick Jones, Mike Moors, Brian Birch, Bhaskar K. Somani

**Affiliations:** 1 Department of Urology, University Hospital Southampton NHS Trust, Southampton, SO16 6YD UK; 2 Primary Care and Population Sciences, University of Southampton, Southampton, UK

**Keywords:** UTI, Urinary infection, Recurrent, Intravesical antibiotics, Antimicrobial resistance

## Abstract

**Purpose of Review:**

Intravesical antibiotics (IVA) has been used for prophylaxis and treatment of recurrent urinary tract infections (rUTIs). However, there is a lack of comprehensive evidence and consensus on its use. We conducted a systematic review to collect all available data about the effectiveness of IVA in prevention and treatment of rUTIs and to give an overview on the outcomes to date.

**Methods:**

A systematic review was carried out for all English language articles from inception to August 2017, according to the Cochrane and PRISMA standards using MEDLINE, Scopus, Biomed Central, EMBASE, CINAHL, and Web of Science with references cross-checked and individual urology journals hand-searched.

**Results:**

After an initial identification of 658 studies, we screened 37 abstracts and 18 full-text papers of which 11 were included in our final review. This included 285 patients with a mean age of 52 years and a female:male ratio of 129:117. The IVA used was gentamicin, neomycin/polymyxin, neomycin or colistin and IVA was used for rUTIs as prophylaxis in 5 studies (*n* = 168) and treatment in 6 studies (*n* = 117). Overall, a good reduction in symptomatic UTI was seen in 78%, with a short-term success rate and discontinuation rates of 71% (120/168) and 8% (14/168) in the prophylaxis group and 88% (103/117) and 5% (6/117) in the treatment groups respectively. There was a change in the sensitivity of organisms in 30% (50/168) and 23% (27/117) in the treatment and prophylaxis groups respectively. Twenty patients discontinued their IVA instillations which were higher for the non-gentamicin group (11%) compared to the gentamicin group (5%). The side effects were minor and included allergy, suprapubic discomfort, autonomic dysreflexia, urinary tract infections and diarrhoea.

**Summary:**

Intravesical antimicrobial instillation seems to be a relatively safe and effective method for the prophylaxis and treatment of recurrent UTIs, especially in the short term. It gives clinicians an alternative treatment modality in high-risk patients predisposed to UTIs where all other forms of systemic treatments have failed.

## Introduction

Instillation of therapeutic agents into the bladder to combat recurrent urinary tract infections (rUTIs) has been part of clinical practice since the 1960s [1, 2, 3•, 4–7, 8•, 9–11]. These are usually reserved as end of line strategy. The inexorable rise in antimicrobial resistance (AMR) has demanded an urgent need for novel solutions for treatment of rUTIs.

Inappropriate use of broad-spectrum antibiotic treatments is considered to have largely contributed to this era of high resistance patterns [12]. Infections associated with such virulent pathogens have become increasingly difficult to manage and often require higher doses or alternative medications. With a paucity of new antibiotics on the horizon, changing the route or regimen of current antibiotic use has become the subject of increased attention. A potential way forward has been the use of intravesical antibiotics (IVA), which have been shown to have greater effect on bacteria at a local level while reducing systemic absorption and its associated side effects [13–16].

The aim of our systematic review was to collate available evidence on the effectiveness of IVA in the prevention and treatment of recurrent urinary infections and to give an overview of the available literature to date.

## Material and Methods

Evidence acquisition: criteria for including studies for this review

Inclusion criteria:

- All English language articles (reporting ≥ 3 patients) of all age groups including paediatric studies
- Studies reporting on IVA for prevention and treatments of rUTIs

Exclusion criteria:

- Case reports, review articles, animal and laboratory studies
- Studies reporting on non-antibiotic intravesical instillations

## Search Strategy and Study Selection

The systematic review was performed according to the Cochrane and preferred reporting items for systematic reviews and meta-analyses (PRISMA) standards [17]. The search strategy was conducted to find all relevant abstracts and publications about bladder antimicrobial instillations for rUTI.

The databases searched included MEDLINE, Scopus, Biomed Central, EMBASE, CINAHL and Web of Science with references cross-checked and individual urology journals hand-searched. The search strategy was conducted to find all relevant abstracts regarding ‘recurrent urinary tract infection’, ‘UTI’, ‘intravesical’, ‘antimicrobial bladder irrigation’, ‘intravesical antimicrobial therapy’, ‘bladder irrigation’, ‘antibiotic intravesical treatment’, ‘instillation’, ‘treatment’, ‘prophylaxis’, ‘recurrent cystitis’ and ‘methods of bladder irrigations’. Boolean operators (AND, OR) were employed to augment the search. The research was limited to English language articles from inception to August 2017. The list of studies generated by the search was screened to identify eligible studies.

### Data Extraction and Outcomes of Interest

The data extraction was carried out by two authors (AP and BS) and any discrepancy was resolved by mutual consensus. Authors were contacted directly in cases of missing data or uncertainty. Primary outcomes of interest were successful treatment or prevention of rUTIs. Data was also collected on method of IVA delivery, follow-up, any change in AMR sensitivity, discontinuation from treatment and the IVA schedule and dose used. While success was defined as reduction in culture positive or symptomatic UTIs, some studies also looked at the change in antibiotic sensitivity with the use of IVA. Due a lack of trials, only pooled analysis of mean results and narrative descriptions have been carried out.

## Results

**Fig. 1:**
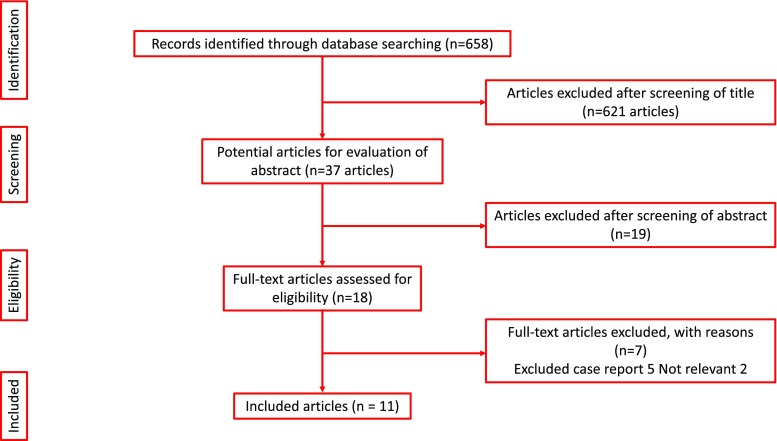
PRISMA flowchart of the included studies

After an initial identification of 658 studies, 37 abstracts were screened followed by review of 18 full text articles (Fig. 1). Eleven of these were included in our final review. This included 285 patients, of which 168 (6 studies, 59%) had IVA as a treatment of rUTI and 117 (5 studies, 41%) had IVA as prophylaxis for rUTI. Except one trial with only 30 participants, all other studies were observational studies [6].

### Baseline Population Characteristics (Table 1)

The mean age participants in the included studies were 52 years with a male to female ratio of 1:1 (Table 1). Although majority of studies (7/11) used gentamicin as the IVA, this included 3 where it was used as treatment and 4 where it was used a prophylaxis against rUTI [2, 4, 5, 8••, 9, 10•, 11]. The other IVA used were a combination of neomycin/polymyxin (*n* = 2) and isolated neomycin and colistin in one study each [1, 3•, 6, 7]. The majority of participants had neurogenic bladders, indwelling catheters or performed intermittent self-catheterisation (ISC), although studies also had patients with urinary diversion (Table 2). All participants had rUTIs when oral antibiotic therapy had failed.

**Table 1:** Characteristics of the selected studies

	Journal	Authors	Intravesical antibiotic	Publication date	Type of study	*N* of patients	Mean age	Underlining pathology	Male:female	Author’s definition of recurrent UTI
Treatment for recurrent UTI										
1	*The Journal of Spinal Cord medicine*	Todd et al.	Neomycin/polymyxin	2016	Retrospective	12	NA	SCI with indwelling catheter	7:3	Failed response to oral antibiotic (2 courses)
2	*The Journal of Urology*	Defoor et al.	Gentamicin	2006	Retrospective	80	10	Neuropathic bladder, bladder exstrophy, cloacal anomalies, Hinman syndrome, vesicoureteral reflux, hypospadias, posterior urethral valves, bladder reconstruction, renal transplantation	38:42	Failed response to oral antibiotic (2 courses)
3	*Infection*	Giua et al.	Colistin	2013	Retrospective	3	75	SCI, mechanical ventilation	2:1	Failed response to oral antibiotic (2 courses)
4	*Infect Urol*	Arap et al.	Gentamicin	2003	Retrospective	18	70	Recurrent UTI	0:18	Failed response to oral antibiotic (2 courses)
5	*The Journal of Urology*	McGuire et al.	Gentamicin	1987	Case series	4	67	Bladder dysfunction, high residual	0:4	Failed response to oral antibiotic (2 courses)
Prophylaxis for recurrent UTI										
6	*The Journal of Spinal Cord medicine*	Waites et al.	Neomycin/polymyxin	2006	Randomised	30	NA	SCI, neurogenic bladder with indwelling catheter or SPC	NA	Recurrent microscopic bacteriuria and pyuria
7	*Antimicrobial Agents and Chemotherapy*	Haldorson et al.	Neomycin	1978	Prospective	53	NA	SCI, vascular disease, MS, cancer	33:20	Recurrent bacteriuria during intermittent catheterisation
8	*Neurourology and Urodynamics*	Abrams et al.	Gentamicin	2017	Retrospective	27	55	Neobladders, neurophatic bladder, ileocystoplasty, ISC	7:20	Recurrent UTI, failed oral prophylaxis
9	*Translational Andrology and Urology*	Dray VE et al.	Gentamicin	2017	Retrospective	22	NA	Patients who required ISC	NA	4 UTIs in the preceding 6-month period
10	*Canadian Urological Association Journal*	Cox et al.	Gentamicin	2017	Prospective	22	37.5	SCI, MS, myelodysplasia transverse myelitis	22:15	4 UTIs in the preceding 6-month period
11	*Meeting of the Infectious Diseases Society of America*	Stalenhoef et al.	Gentamicin	2013	Retrospective	14	60	ISC, chronic bacterial prostatitis, vesicoureteral reflux and neobladder	8:6	Recurrent UTI despite, failed oral prophylaxis

*NA* not available, *SCI* spinal cord injury, *ISC* intermittent self-catheterisation, *MS* multiple sclerosis

**Table 2:** Characteristics of the intravesical therapy

	Intravesical antibiotic	Administration	Serum level	Successful outcome*	Follow-up (range)	Change in sensitivity**	Unchanged resistance	Discontinuation	Reasons
Treatment for recurrent UTI									
1	Neomycin/polymyxin	Staff	Not checked	9/12 (75%)	6 months (no range)	9/12 (75%)	3/12 (25%)	2/12 (16%)	Allergy
2	Gentamicin	Parents	Yes negligible	75/80 (93.7%)	90 days (3–1095)	16/80(20%)	5/80 (6%)	Variable not trackable	0
3	Colistin	Staff	Not checked	3/3 (100%)	NA	2/3 (66%)	0/3	1/3 (33%)	1 suprapubic discomfort
4	Gentamicin	ISC	Yes negligible	12/18 (66%)	65.1 months (15–103)	0	3/18 (16%)	3/18 (16%)	UTI
5	Gentamicin	ISC	Not checked	4/4 (100%)	22 weeks (18–24)	0	0	0	0
Overall treatment outcomes				103/117 (88%)		27/117 (23%)	11/117 (9%)	6/117 (5%)	
Prophylaxis for recurrent UTI									
6	Neomycin/polymyxin	Self	Not checked	23/30 (76%)	8 weeks (no range)	19/30 (63%)	7/30 (23%)	8/30 (26%)	4 UTIs, 4 other health-related issues
7	Neomycin	Staff	Not checked	16/53 (30%)	6 weeks (1–19)	NA	37/53 (70%)	0	NA
8	Gentamicin	Self	Yes negligible	27/27 (100%)	24 months (2–67)	18/27 (66%)	1/27 (3%)	6/27 (22%)	Infection, stones, cystectomy, nephrectomy, bladder stone
9	Gentamicin	Self/carer	Yes negligible	22/22 (100%)	NA	NA	0	NA	NA
10	Gentamicin	Self	Not checked	22/22 (100%)	6 weeks (no range)	9/22 (40%)	8/22 (36%)	0	0
11	Gentamicin	Self	Yes negligible	10/14 (71%)	42 weeks (6–148)	4/14 (28%)	2/14 (14%)	0	0
Overall treatment outcomes				120/168 (71%)		50/168 (30%)	55/168 (32%)	14/168 (8%)	

*NA* not available

*Successful outcome is met in those where the irrigation procedure had to eradicate all of the organisms or change the sensitivities so oral antibiotics could be given

**Resistant organism was eradicated, or the sensitivity changed to one that could be treated with an oral antibiotic

### Outcomes

The majority of the participants who underwent the antimicrobial instillation showed a good response with a reduction of symptomatic UTI in 78.2% (*n* = 223) (Table 2). A high success rate of 88% (*n* = 103%) was seen in the treatment group in the short term (3–6 months) [1, 2, 3•, 4, 5], with a low discontinuation rate of 5% (*n* = 6). A success rate of 71% (*n* = 120) was seen in the prophylaxis group in the short term [6, 7, 8••, 9, 10•, 11], with a discontinuation rate of 8% (*n* = 14).

### Effect of IVA

Overall, there was a change in the sensitivity of organisms in 30% (*n* = 50) and 23% (*n* = 27) in the treatment and prophylaxis groups respectively, which meant that either the resistant organism was eradicated or the sensitivity changed so that further treatment could be carried out with oral antibiotics.

Overall, 20 patients discontinued their IVA instillation (6 in the treatment group, 14 in the prophylaxis group). The gentamicin group had a discontinuation rate of 5% (*n* = 9), which was 3% (*n* = 3) for treatment group and 7% (*n* = 6) for the prophylaxis group. The non-gentamicin group had a discontinuation rate of 11% (*n* = 11), which was 20% (*n* = 3) for the treatment group and 10% (*n* = 8) for the prophylaxis group.

While the side effects of treatment were not well documented in studies, most reported were minor and included allergy, suprapubic discomfort, autonomic dysreflexia, urinary tract infections and diarrhoea (Table 3). A slight increase in serum levels of gentamicin was also seen in 4% (*n* = 3) in one series (2).

**Table 3:** Dose, duration and side effects of antibiotics used for intravesical treatment

	Drug	Dose	Duration	Side effect
Treatment for recurrent UTI				
1	Neomycin/polymyxin	30 ml	3/day for 5 days	Allergy
2	Gentamicin	0.48 mg/ml in 30 ml normal saline	2/day	Minor rise in serum creatinine for 3 patients with chronic renal insufficiency
3	Colistin	3.5 mg 0.42% dissolved in 500 ml of saline solution	3/day for 7 days	1 suprapubic discomfort
4	Gentamicin	480 mg + 1 l saline solution+100 ml sodium carbonate	30 ml daily for 1 week, then alternate days for 6 weeks (2 months)	3 UTI
5	Gentamicin	20 ml of 240 mg in 1 l saline solution	4 ISC/day	0
Prophylaxis for recurrent UTI				
6	Neomycin/polymyxin	30 of 40 mg/ml neomycin sulfate and 200,000 units/ml polymyxin B	2/day for 8 weeks	2 autonomic dysreflexia
7	Neomycin	0.1% of neomycin solution	After each intermittent catheterisation	nd
8	Gentamicin	80 mg in 10 ml saline solution	Nightly	0
9	Gentamicin	14.4–28.8 mg in 30–60 ml of saline solution	Nightly	0
10	Gentamicin	14.4–28.8 mg in 30–60 ml of saline solution	Nightly	1 yeast infection, 1 diarrhoea
11	Gentamicin	80 mg in 20 ml of saline solution	Daily then once a week	0

*UTI* urinary tract infection, *ISC* intermittent self-catheterisation, *nd* no data

## Discussion

### Meaning of the Study

This is the first review of its kind on the role of IVA for prophylaxis and treatment of rUTIs. The overall short-term success rate seems to be good, in regard to both its role in prophylaxis and treatment. It is associated with low risk of complications and discontinuation rates of between 5 and 8%.

### Role of IVA

The earlier reports on IVA date back to 1967 when most studies were case reports. It was in 1996 that Hajjar et al. described a strain of resistant bacteria due to indiscriminate antibiotic use and reported a case of vancomycin bladder irrigation (via a three-way catheter) to treat MRSA [18]. Then, in 1978, Haldorson et al. tested the use of neomycin in reducing bacteriuria in a case-control group after ISC [7]. In 2004, Wood et al. described a successful case of tobramycin bladder irrigation for UTI in a critically ill patient [14]. Since then, other studies about intravesical agents have been described with wider cohorts of patients.

Other non-antimicrobial intravesical instillations such as hyaluronic acid and chondroitin sulfate have also been used [15]. During our literature research, we also identified other antimicrobials such as tobramicin, linezolid and vancomycin which have been used as IVA. However, these results were mostly limited to isolated case reports [12, 14, 18].

The majority of patients with rUTIs, either from idiopathic UTI or due to an underlining pathology, often have a poor quality of life. In the presence of underlying risk factors such as spinal cord injury (SCI), urinary diversion or intermittent self-catheterisation (ISC) [2, 3•, 4–7, 8••, 9, 10•, 11], it is marked by frequent hospital admissions with repeated use of wide-spectrum intravenous antibiotics [13]. However, over time, there is development of both bacterial resistance and the systemic side effects that are well recognised after protracted courses of intravenous and oral antibiotics [15]. Waites and colleagues also demonstrated that oral antibiotics change the urinary, perineal and urethral flora of neurogenic bladder patients [6].

While gentamicin was the most common IVA used, the dosage varied in different studies [8••, 12]. Wan et al. studied the safety and efficacy of intravesical gentamicin instillations in rat models [19]. They demonstrated that although severe bladder inflammation and anatomical abnormalities can increase transvesical absorption of gentamicin, serum gentamicin levels were still in the therapeutic range. Canine models showed that despite the presence of vesicoureteric reflux (VUR), serum gentamicin levels were not detectable after intravesical instillation [19, 20]. Furthermore, they also studied 10 children who were performing ISC for neurogenic bladder dysfunction, and none had detectable levels of serum gentamicin at 30-min post-instillation, with no adverse reactions noted [19]. A similar test was carried out by Defoor et al. in 80 paediatric patients and none of them were found to have a serum gentamicin level greater than 0.4 μg/ml [2]. Small increases in serum creatinine were seen in 3 patients with chronic renal insufficiency. However, this was believed to be attributable to the progression of the native renal disease.

IVA not only seems to decrease the frequency of symptomatic infections, but potentially seems to play a role in reducing the need for oral antibiotics. While gentamicin seems to be more widely used and has shown to be effective in the bladder, other IVA have not had a similar response and currently seem to have insufficient clinical evidence due to a lack of adequate published data on them [8••].

### Limitations and Areas of Future Research

Our review is limited by the observational and retrospective nature of most studies and limited data for management of end of line rUTIs. Only one small randomised trial was identified [6]. Although the majority of studies used gentamicin, not all did so and both dosage and schedules were not standardised. The gentamicin dose used varied from 14.4 to 480 mg mixed in saline with no fixed treatment duration recommended for it. This is likely due to the rarity of this treatment and uncertainty amongst clinicians and microbiologists as to the most appropriate regimen. The systematic side effects of IVA perhaps have not fully been explored [21], but intermittent, single-dose regimens are unlikely to have systemic side effects. The role of intravesical non-antibiotic treatments for rUTIs also needs to be better defined as there is some data to support this [22].

Although the standardisation of IVA treatment will be a topic of future research, patient compliance and long-term follow-up will be required to establish the true benefit in patients with rUTIs. Despite methodological limitations, given the rarity of this treatment, the potential benefit of IVA for prophylaxis and treatment of patients with rUTIs cannot be ignored. It is therefore important to have protocol-based collaborative centre studies supported by a large sample size to establish the role of IVA in these patients. Ideally, this needs to be addressed by an RCT with a placebo or non-antibiotic comparator, evaluating not only the treatment outcomes but also on the drug dosages, instillation regime, quality of life and cost associated with it.

## Conclusions

Intravesical antimicrobial instillation seems to be a relatively safe and effective method for the prophylaxis and treatment of recurrent UTIs, especially in the short term. It gives clinicians an alternative treatment modality in high-risk patients predisposed to UTIs where all other forms of systemic treatments have failed.

## References

[1] Todd A, Anuj J, Thompson W (1999). Effectiveness of neomycin/polymyxin bladder irrigation to treat resistant urinary pathogens in those with spinal cord injury. J Spinal Cord Med.

[2] Defoor W, Ferguson D, Mashni S, Creelman L, Reeves D, Minevich E, Reddy P, Sheldon C (2006). Safety of gentamicin bladder irrigations in complex urological cases. J Urol.

[3] Giua R, Pedone C, Cortese L (2014). Colistin bladder instillation, an alternative way of treating multi-resistant Acinetobacter urinary tract infection: a case series and review of literature. Infection.

[4] Arap M, Petrou PS. Efficacy of intermittent intravesical gentamicin sulfate solution for recalcitrant recurrent cystitis in women. Infect Urol. 2003;16(2)

[5] McGuire EJ, Savastano JA (1987). Treatment of intractable bacterial cystitis with intermittent catheterization and antimicrobial instillation: case report. J Urol.

[6] Waites KB, Canupp KC, Roper JF (2006). Evaluation of 3 methods of bladder irrigation to treat bacteriuria in persons with neurogenic bladder. J Spinal Cord Med.

[7] Haldorson AM, Keys TM, Maker MD (1978). Non value of neomycin instillation after intermittent urinary catheterization. Antimicrob Agents Chemother.

[8] Abrams P, Hashim H, Tomson C, Macgowan A, Skews R, Warren K (2017). The use of intravesical gentamicin to treat recurrent urinary tract infections in lower urinary tract dysfunction. Neurourol Urodyn.

[9] Dray VE, Clemens JQ (2017). Recurrent urinary tract infections in patients with incomplete bladder emptying: is there a role for intravesical therapy?. Transl Androl Urol.

[10] Cox L, He C, Bevins J (2017). Gentamicin bladder instillations decrease symptomatic urinary tract infections in neurogenic bladder patients on intermittent catheterization. Can Urol Assoc J.

[11] Stalenhoef EJ, Van Nieuwkoop C, Elzevier HW, et al. Intravesical gentamicin for symptomatic recurrent urinary tract infection caused by multidrug-resistant microorganisms. Meeting of the Infectious Diseases Society of America. (2013). https://idsa.confex.com/idsa/2013/webprogram/Paper41056.html.

[12] Alfthan O, Renkonen OV, Sivonen A (1973). Concentration of gentamicin in serum, urine, and urogenital tissue in man. Microbiol Immunol.

[13] Hill DM, Wood GD, Hickerson WL (2015). Linezolid bladder irrigation as adjunctive treatment for a vancomycin-resistant *Enterococcus faecium* catheter-associated urinary tract infection. Ann Pharmacother.

[14] Wood GC, Chapman JL, Boucher BA, Mueller EW, Fabian TC, Croce MA (2004). Tobramycin bladder irrigation for treating a urinary tract infection in a critically ill patient. Ann Pharmacother.

[15] Bergamin AP, Kiosoglous AJ (2017). Non-surgical management of recurrent urinary tract infections in women. Transl Androl Urol..

[16] De Jong P, Donckerwolcke R, Boemers TM (1993). Neomycin toxicity in bladder irrigation. J Urol.

[17] Higgins, JPT, Green S (editors). *Cochrane handbook for systematic reviews of interventions* Version 5.1.0 [updated March 2011]. The Cochrane Collaboration, 2011. Available from http://handbook.cochrane.org.

[18] Hajjar RR, Philpot C, Morley JE (1996). Continuous bladder irrigation with vancomycin for the treatment of methicillin-resistant *Staphylococcus aureus*. J Am Geriatr Soc.

[19] Wan J, Kozminski M, Wang SC, Faerber GJ, McGuire EJ, Bloom DA, Ritchey ML (1994). Intravesical instillation of gentamicin sulfate: in vitro, rat, canine, and human studies. Urology.

[20] Van Nieuwkoop C, Den Exter PL, Elzevier HW (2010). Intravesical gentamicin for recurrent urinary tract infection in patients with intermittent bladder catheterisation. Int J Antimicrob Agents.

[21] Gerharz EW, Weingärtner K, Melekos MD (1995). Neomycin-induced perception deafness following bladder irrigation in patients with end-stage renal disease. Br J Urol.

[22] Torella M, Schettino MT, Salvatore S (2013). Intravesical therapy in recurrent cystitis: a multi-center experience. J Infect Chemother.

